# Differences in mutational signature of diffuse large B‐cell lymphomas according to the primary organ

**DOI:** 10.1002/cam4.6533

**Published:** 2023-09-14

**Authors:** Hyun‐Hee Koh, Sang Eun Yoon, Seok Jin Kim, Won Seog Kim, Junhun Cho

**Affiliations:** ^1^ Department of Pathology, Samsung Medical Center Sungkyunkwan University School of Medicine Seoul Korea; ^2^ Department of Pathology, Severance Hospital Yonsei University College of Medicine Seoul Korea; ^3^ Division of Hematology and Oncology, Department of Internal Medicine Sungkyunkwan University School of Medicine Seoul Korea

**Keywords:** breast, diffuse large B‐cell lymphoma, ileocecal area, mutational signature, primary organ

## Abstract

**Background:**

Comprehensive molecular subtyping of diffuse large B‐cell lymphoma (DLBCL) through genetic profiling has broadened our understanding of DLBCL biology. In this study, we investigated whether DLBCL, not otherwise specified (NOS) shows differences in mutational patterns depending on the primary organ.

**Patients and Methods:**

Panel‐based next‐generation sequencing was performed on 345 DLBCL from various primary organs, and patterns of mutations according to primary organs were analyzed.

**Results:**

DLBCL showed a characteristic mutational signature in several primary organs. Among them, the mutational pattern of DLBCL in the breast and ileocecal area was particularly different from that of other DLBCL NOS. In breast DLBCL, *MYD88* ^L265P^ (57.1%), *CD79B* mutation (42.9%), and *CDKN2A*/*B* loss (71.4%) were found at high frequencies, which were similar to the mutation patterns of DLBCL of immune‐privileged sites compared with DLBCL NOS. DLBCL in the ileocecal area showed a characteristic mutation pattern with the most frequent *TP53* mutation (52.6%) and 18q21 gain (42.1%). This was also different from the mutational pattern observed in the stomach or other intestines. In discriminant analysis, DLBCL of the breast and ileocecal area tended to form separate genetic constellations from other DLBCL NOS.

**Conclusion:**

DLBCL NOS has a characteristic mutational profile that depends on the primary organ. In particular, the mutational signature of DLBCL in the breast and ileocecal area was heterogeneous compared with that of other DLBCL NOS. Further research is needed to determine whether primary DLBCL in the breast and ileocecal area can be classified as an independent subtype.

## 1 INTRODUCTION

Diffuse large B‐cell lymphoma (DLBCL) is a heterogeneous entity. Numerous studies have been conducted to classify specific subtypes of DLBCL, and recently the updated World Health Organization (WHO) classification^1^ and The International Consensus Classification (ICC)^2^ have suggested a more subdivided categorization of DLBCL. DLBCL is thought to be caused by differences in pathogenesis not only by the cell of origin^3^, ^4^, ^5^, ^6^ but also by special microenvironments such as immune‐privileged sites,^7^, ^8^ indigenous cells such as asteroid B‐cells in the mediastinum, and clinical settings such as chronic inflammation or Epstein–Barr virus infection. A number of DLBCL cases are still diagnosed as not otherwise specified (NOS), which may also be a set of members of a particular subtype that has not yet been classified.

Recent breakthroughs in molecular biology have allowed DLBCL NOS to be classified into more detailed groups. Chapuy et al. classified DLBCL into five molecular subtypes from C1 to 5 through comprehensive genetic analysis,^9^ and Wright et al. divided DLBCL into seven genetic subtypes: MCD, BN2, EZB (MYC+ and MYC−), ST2, A53, and N1 and presented a tool for their probabilistic classification.^10^, ^11^ The proposed classification schemes for subdividing DLBCL are expected to help identify pathophysiology, predict accurate prognosis, and develop individualized targeted therapy.^12^, ^13^ In a clinical trial, it was reported that the therapeutic effect of Bruton tyrosine kinase inhibitor combined with R‐CHOP (rituximab, cyclophosphamide, doxorubicin, vincristine, and prednisone) was greater in MCD and N1 subtypes aged less than 60 years compared with other subtypes.^14^ This suggests the possibility that more targeted therapies through molecular subtyping of DLBCL can be applied to the treatment of actual patients in the future.

Many of the established subtypes of DLBCL, such as primary large B‐cell lymphoma of the central nervous system (PCNSL), primary large B‐cell lymphoma of the testis (PTL), and primary mediastinal large B‐cell lymphoma (PMLBL), are named according to their primary organs. In addition to these, there have been studies on the characteristic genetic mutations of DLBCL that occurred in the bone,^15^ breast,^16^, ^17^ and gastrointestinal (GI) tract^18^, ^19^, ^20^ among DLBCL NOS. Additionally, there were reports that Helicobacter pylori (H. pylori) eradication was reported to be effective in early‐stage H. pylori‐positive gastric DLBCL^21^, ^22^ and studies showed that there was a survival benefit to performing chemotherapy after surgery rather than chemotherapy alone in intestinal DLBCL.^23^, ^24^ As such, different therapeutic strategies are sometimes applied to DLBCL in a specific site, suggesting that there may be differences in the tumor biology of DLBCL depending on the primary organ. With this in mind, we analyzed the panel‐based next‐generation sequencing (NGS) results of 345 DLBCLs according to the primary organ and found that DLBCL NOS occurring in some primary organs, such as the breast and ileocecal (IC) area, showed characteristic mutational signatures.

## 2 PATIENTS AND METHODS

### 2.1 Patient selection

Among patients diagnosed with malignant lymphoma at Samsung Medical Center, Seoul, Korea, between January 2019 and May 2022, 345 consecutive DLBCL cases in which panel‐based NGS (LymphomaSCAN) was performed were included in this study. The patient's clinical and pathological information was reviewed using the electronic medical record system. There were 211 males and 134 females, with a male‐to‐female ratio of 1.57:1. The median age of the patients was 61 years (range, 20–90 years). According to the Ann Arbor stage, 223 (64.6%) were stage I‐II and 122 (35.4%) were stage III‐IV. By diagnosis, there were 296 (85.8%) patients of DLBCL NOS, 28 (8.1%) patients of PCNSL, 12 (3.5%) patients of PMLBL, and 9 (2.6%) patients of PTL. Among DLBCL NOS, 110 (37.5%) were germinal center B‐cell (GCB) and 183 (62.5%) were non‐GCB according to the Hans algorithm.^25^ Primary organs of 296 DLBCL NOS cases were lymph node in 78 (26.4%), nasal cavity/nasopharynx in 16 (5.4%), tonsil in 23 (7.8%), GI tract in 48 (16.2%) (stomach in 17 [5.7%], IC area in 19 [6.4%], and other intestines in 12 [4.0%]), and breast in 7 cases (2.4%). Thirty (10.1%) cases with five or fewer tumors in one organ were separately classified as the other group. In 94 (31.6%) patients, the primary site could not be identified because tumors were found in multiple organs at the time of initial diagnosis; therefore, they were classified as the unknown group. The clinical and pathological information of the patients is summarized in Table 1. All methods were performed in accordance with the Helsinki Declaration, and the Institutional Review Board of Samsung Medical Center approved all protocols of this study (IRB file number: 2021‐01‐093‐004). A waiver of written informed consent was granted by the Institutional Review Board.

**TABLE 1:** Clinical and pathological characteristics of 345 patients.

Characteristics	Number	%
Sex		
Male	211	61.2%
Female	134	38.8%
Diagnosis		
DLBCL NOS	296	85.8%
PCNSL	28	8.1%
PTL	9	2.6%
PMLBL	12	3.5%
Ann Arbor stage		
I‐II	223	64.6%
III‐IV	122	35.4%
Cell of origin (in NOS)		
GCB	110	37.5%
Non‐GCB	183	62.5%
N/A	3	
Ann Arbor stage		
I‐II	223	64.6%
III‐IV	122	35.4%
Primary site (in NOS)		
Lymph node	78	26.4%
Head and neck	39	
NC/NPhx	16	5.4%
Tonsil	23	7.8%
GI tract	48	
Stomach	17	5.7%
IC area	19	6.4%
Other intestines	12	4.0%
Breast	7	2.4%
Other sites	30	10.1%
Unknown	94	31.8%

Abbreviations: DLBCL, diffuse large B‐cell lymphoma; GCB, germinal center B‐cell; GI, gastrointestinal; IC, ileocecal; NC/NPhx, nasal cavity/nasopharynx; NOS, not otherwise specified; PCNSL, primary large B‐cell lymphoma of the central nervous system; PMLBL, primary mediastinal large B‐cell lymphoma; PTL, primary large B‐cell lymphoma of the testis.

### 2.2 Panel‐based next‐generation sequencing

Targeted genetic sequencing was performed, and in this study, single nucleotide variant (SNV) of 44 genes and copy number variant (CNV) of 11 genes or regions were used for analysis based on previous studies.^1^, ^2^, ^9^, ^11^, ^26^, ^27^, ^28^ (Tables S1 and S2). Extracted genomic DNA was sheared using a Covaris S220 (Covaris). Targeted genes were captured using the SureSelect XT Reagent Kit, HSQ (Agilent Technologies), and a paired‐end sequencing library was constructed using a barcode. DNA sequencing was performed on a NextSeq 550 Dx sequencer (Illumina). Paired‐end reads were aligned to the human reference genome (hg19) using BWA‐MEM v0.7.5, Samtools v0.1.18, GATK v3.1‐1, and Picard v1.93. SNVs were called using MuTect version 1.1.4, LoFreq version 0.6.1, and VarDict version 1.06 software with a variant allele frequency (VAF) ≥1% or a number of variant supporting reads >4. We manually reviewed variants with supporting reads <20 using an Integrative Genomics Viewer browser and filtered out sequencing errors. We identified small Indels using Pindel version 0.2.5a4 with a number of variant supporting reads >9. We further filtered out variants with a minor allele frequency ≥1% from the 1000 Genomes Project database,^29^ the Exome Aggregation Consortium database,^30^ the National Heart, Lung, and Blood Institute's Exome Sequencing Project database,^31^ the Korean Reference Genome Database,^32^ the Korean Variant Archive,^33^ and an in‐house database of 192 Korean individuals. To consistently measure the number of mutations, only SNV/Indel results were used, whereas copy number variation and fusion results were discarded. To filter out false‐positive results, variants with a VAF <5% and total reads <100 were excluded. The multiplicity of mutations found in a single gene was not considered as a variable in this study.

### 2.3 Statistical analysis

We used SPSS 27 (IBM Corporation) and R software programs for statistical analyses. The differences in mutation frequency between each group were tested using chi‐squared test, and the *p*‐value was calculated using the false discovery rate to control for type I error in multiple comparisons using the Benjamini–Yekutieli method. *p*‐values <0.05 were considered statistically significant. Multiple discriminant analysis using independent variables together method was performed to classify the tumors of each original organ according to molecular characteristics.

## 3 RESULTS

### 3.1 The overall distribution of genetic alterations

The frequency of SNV in all DLBCL (*n* = 345) was *PIM1* (*n* = 137, 39.7%), *CD79B* (*n* = 96, 27.8%), *HIST1H1E* (*n* = 89, 25.8%), *TP53* (*n* = 87, 25.2%), *BTG1* (*n* = 86, 24.9%), and *MYD88* ^L265P^ (*n* = 84, 24.3%; Table S1). The frequency of CNV in all cases was *CDKN2A*/*B* loss (*n* = 96, 27.8%), 18q21 (including *BCL2*, *MALT1*, *SMAD2*, *SMAD4*, and *TNFRSF11A*) gain (*n* = 43, 12.5%), and *REL* gain (*n* = 19, 5.5%; Table S2). Among DLBCL NOS (*n* = 296), the frequency of SNV was *PIM1* (*n* = 101, 34.1%), *TP53* (*n* = 79, 26.7%), *CD79B* (*n* = 72, 24.3%), *BTG1* (*n* = 71, 24.0%), and *HIST1H1E* (*n* = 70, 23.6%). The frequency of CNV in DLBCL NOS was *CDKN2A*/*B* loss (*n* = 72, 24.3%) and 18q21 gain (*n* = 40, 13.5%). Among GCB‐type DLBCL NOS (*n* = 110), the frequency of SNV was as follows: *PIM1* (*n* = 34, 30.9%), *TP53* (*n* = 31, 28.2%), *BTG1* (*n* = 25, 22.7%), *HIST1H1E* (*n* = 24, 21.8%), and *EP300* (*n* = 24, 21.8%). The frequency of CNV in GCB‐type DLBCL NOS was *CDKN2A*/*B* loss (*n* = 24, 21.8%) and *REL* gain (*n* = 15, 13.6%). Among the non‐GCB‐type DLBCL NOS (*n* = 183), the frequency of SNV was *PIM1* (*n* = 66, 36.1%), *CD79B* (*n* = 54, 29.5%), *TP53* (*n* = 47, 25.7%), *HIST1H1E* (*n* = 45, 24.6%), and *ETV6* (*n* = 45, 24.6%). The frequency of CNV in non‐GCB‐type DLBCL NOS was *CDKN2A*/*B* loss (*n* = 48, 26.2%) and 18q21 gain (*n* = 29, 15.8%).

### 3.2 Comparison of genetic alterations in DLBCL NOS and specific subgroups

**FIGURE 1:**
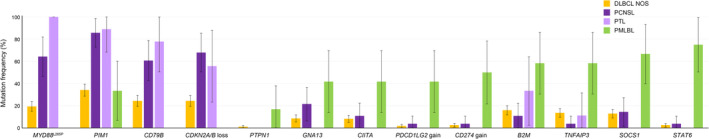
Bar graphs of genes showing statistically significant different mutation frequencies in diffuse large B‐cell lymphoma, not otherwise specified, primary large B‐cell lymphoma of immune‐privileged sites (primary large B‐cell lymphoma of the central nervous system and testis), and primary mediastinal large B‐cell lymphoma. The lines on the bar graphs represent 95% confidence intervals (DLBCL NOS, diffuse large B‐cell lymphoma, not otherwise specified; PCNSL, primary large B‐cell lymphoma of the central nervous system; PMLBL, primary mediastinal large B‐cell lymphoma; PTL, primary large B‐cell lymphoma of the testis).

In patients with PCNSL (*n* = 28), the frequency of SNV was *PIM1* (*n* = 24, 85.7%), *MYD88* ^L265P^ (*n* = 18, 64.3%), and *CD79B* (*n* = 17, 60.7%). The most common CNV in PCNSL was *CDKN2A*/*B* loss (*n* = 19, 67.9%). In PTL (*n* = 9), the frequency of SNV was *MYD88* ^L265P^ (*n* = 9, 100%), *PIM1* (*n* = 8, 88.9%), and *CD79B* (*n* = 7, 77.8%). The most common CNV in PTL was *CDKN2A*/*B* loss (*n* = 5, 55.6%). In PMLBL (*n* = 12), SNV was most frequent in the order of *STAT6* (*n* = 9, 75.0%), *SOCS1* (*n* = 8, 66.7%), *B2M* (*n* = 7, 58.3%), and *TNFAIP3* (*n* = 7, 58.3%), and *MYD88* ^L265P^ and *CD79B* mutations were absent. The most common CNV in PMLBL was *CD274* gain (*n* = 6, 50.0%) and *PDCD1LG2* gain (*n* = 5, 41.7%; Tables S1 and S2). When PCNSL and PTL were grouped as primary large B‐cell lymphoma of immune‐privileged tissue, the genes showing statistically significant differences in DLBCL NOS, immune‐privileged sites, and PMLBL were *STAT6*, *MYD88* ^L265P^, *PIM1*, *CD79B*, *SOCS1*, *TNFAIP3*, *PTPN1*, *CIITA*, *GNA13*, and *B2M* in SNV, and *CD274* gain, *PDCD1LG2* gain, *CDKN2A* loss, and *CDKN2B* loss in CNV (Figure 1).

### 3.3 Comparison of genetic alterations according to the primary organ of DLBCL NOS

Information on mutations according to the primary organ of DLBCL is summarized in Table S3. In DLBCL NOS in the lymph node (*n* = 78), mutations in *PIM1* (*n* = 22, 28.2%), *BTG1* (*n* = 22, 28.2%), and *TP53* (*n* = 20, 25.6%) were the most common. In the nasal cavity/nasopharynx (*n* = 16), *PIM1* (*n* = 8, 50.0%), *CDKN2A*/*B* loss (*n* = 6, 37.5%), *BTG* (*n* = 5, 31.2%), and *ETV6* (*n* = 5, 31.2%) mutations were common. In the palatine tonsil (*n* = 23), *PIM1* (*n* = 15, 65.2%), *BTG1* (*n* = 7, 30.4%), *SGK1* (*n* = 7, 30.4%), and *SOCS1* (*n* = 7, 30.4%) mutations were common. In the breast (*n* = 7), *CDKN2A*/*B* loss (*n* = 5, 71.4%), *MYD88* ^L265P^ (*n* = 4, 57.1%), *PIM1* (*n* = 3, 42.9%), *CD79B* (*n* = 3, 42.9%), *B2M* (*n* = 3, 42.9%), and *CIITA* (*n* = 3, 42.9%) mutations were common. In the stomach (*n* = 17), *BTG1* (*n* = 6, 35.3%), and *CARD11* (*n* = 5, 29.4%) mutations were common. In the IC area (*n* = 19), *TP53* (*n* = 10, 52.6%), and 18q21 gain (*n* = 8, 42.1%) mutations were common. In other intestines (*n* = 12), *TP53* (*n* = 5, 41.7%), and *B2M* (*n* = 4, 33.3%) mutations were common. In DLBCL NOS in other organs (*n* = 30), the most common mutations were *PIM1* (*n* = 13, 43.3%), *MYD88* ^L265P^ (*n* = 12, 40.0%), *TP53* (*n* = 10, 33.3%), and *CD79B* (*n* = 10, 33.3%). In DLBCL NOS of unknown primary organ (*n* = 94), *PIM1* (*n* = 35, 37.2%) and *CDKN2A*/*B* loss (*n* = 27, 28.7%) mutations were common.

**FIGURE 2:**
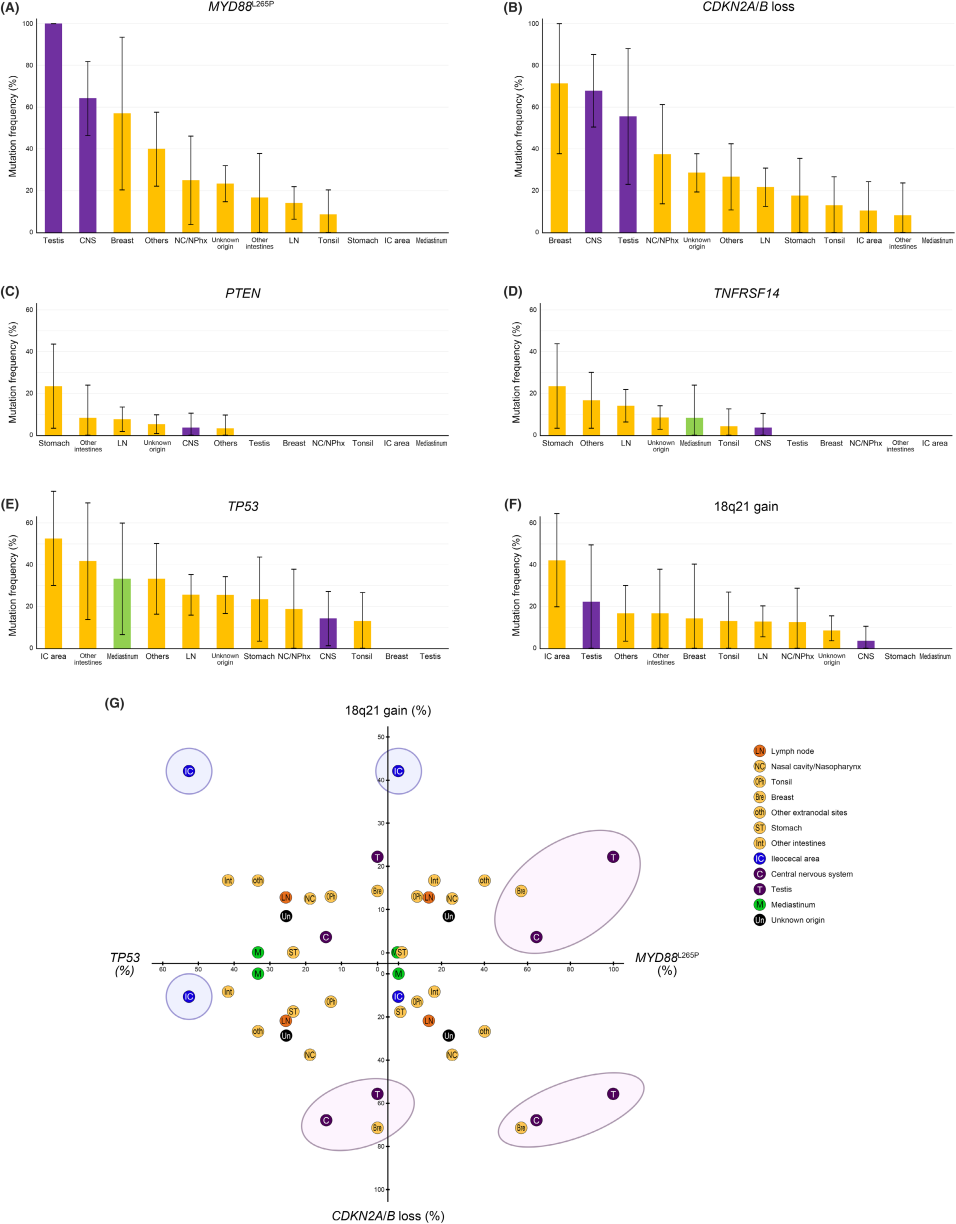
(A–F) Bar graphs of the mutation frequency by primary organ of representative genes. Diffuse large B‐cell lymphoma, not otherwise specified is shown as yellow, primary large B‐cell lymphoma of immune‐privileged sites as purple, and primary mediastinal large B‐cell lymphoma as green bars. The lines on the bar graphs represent 95% confidence intervals. (G) Scatter plot of diffuse large B‐cell lymphomas in each primary organ according to the mutation frequencies of four representative genetic alterations. Tumors originating from the central nervous system (purple), testis (purple), breast (yellow), and ileocecal area (blue) showed characteristic mutation patterns differentiated from the tumors of many other organs (CNS, central nervous system; IC, ileocecal; LN, lymph nodes; NC/NPhx, nasal cavity and nasopharynx).

The frequency of the *MYD88* ^L265P^ mutation was highest at 57.1% in the breast, 40% at other sites, and 14.1% at the lymph node. No *MYD88* ^L265P^ mutation was found in the stomach and IC area (Figure 2A–F). The frequency of the *CD79B* mutation was highest in the breast (42.9%), followed by other sites (30%), and was 26.3%, 23.1%, and 17.6% in the IC area, lymph node, and stomach, respectively. The frequency of *CDKN2A*/*B* loss was highest in the breast (71.4%), 21.8% in the lymph node, and lowest in the other intestines (8.3%) among DLBCL NOS. *PTEN* mutations were most frequent in the stomach at 23.5%, followed by other intestines at 8.3%. No *PTEN* mutations were found in the breast, nasal cavity/nasopharynx, tonsil, or IC area. *TNFRSF14* mutations were most commonly observed in the stomach (23.5%), but not in other intestines, breast, nasal cavity/nasopharynx, or IC area. The frequency of *TP53* mutation was highest in the IC area (52.6%), followed by the other intestines (41.7%). *TP53* mutation in the lymph node and stomach were 25.6% and 23.5%, respectively, and were not detected in the breast. 18q21 gain was most frequently found in the IC area (42.1%), followed by other intestines and other sites (16.7%). The frequency of 18q21 gain in the lymph node was 12.8% and was not found in the stomach. When the tumors that occurred in each organ were depicted as a scatter plot with the frequencies of *MYD88* ^L265P^, *CDKN2A*/*B* loss, *TP53*, and 18q21 gain as four axes, the breast DLBCL was located in a similar location to the PCNSL and PTL, and the DLBCL of the IC area was located far from other DLBCL NOS (Figure 2G).

### 3.4 Discriminant analysis of all DLBCLs according to the primary organ

**FIGURE 3:**
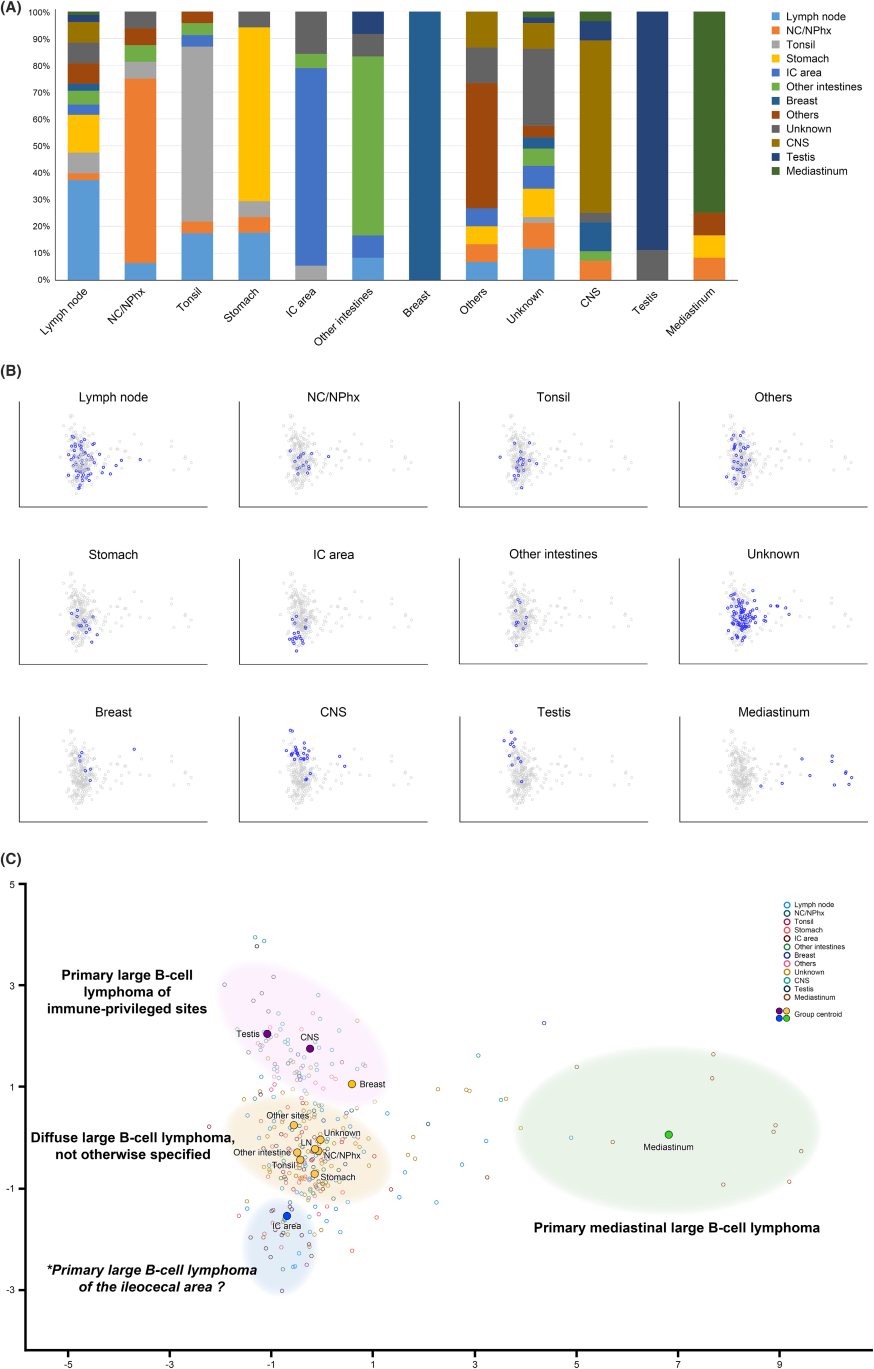
Results of discriminant analysis. (A) Graphs showing the proportion of diffuse large B‐cell lymphoma in the original group classified into which prediction group by primary organ. Single‐colored and multicolored bars represent genetic homogeneity and heterogeneity of tumors, respectively. (B, C) Scatter plots of diffuse large B‐cell lymphoma of various primary organs according to the pattern of genetic variation. Graphs highlighting the distribution of points for each primary organ in the background of all cases are shown in (B). The location of the centroids of dot of each primary organ is depicted in (C). By the location of centroids, the mediastinum, central nervous system, testis, breast, and ileocecal area were out of the constellation of diffuse large B‐cell lymphoma, not otherwise specified.

As a result of discriminant analysis, the concordance between the original and predicted groups was 100% (7/7) in the breast, 88.9% (8/9) in the testis, 75.0% (9/12) in the mediastinum, 73.7% (14/19) in the IC area, 68.8% in nasal cavity/nasopharynx (11/16), 66.7% (8/12) in other intestines, 65.2% (15/23) in the tonsil, 64.7% (11/17) in the stomach, 64.3% (18/28) in the CNS, 46.7% (14/30) in other sites, 37.2% (29/78) in the lymph node, and 28.7% (27/94) in unknown primary organs (Table 2 and Figure 3A). Scatter plots according to discriminant analysis are shown in Figure 3B,C. According to the centroid spots, the mediastinum had the most different mutational profile from the other DLBCLs. The testis and CNS also appeared to have a different mutational profile from DLBCLs in other organs. Breast DLBCL, currently classified as DLBCL NOS, formed centroids in locations similar to immune‐privileged sites rather than DLBCL NOS. The IC area DLBCL was also different from many other DLBCLs, and the centroid was located far from the CNS, testis, and breast.

**TABLE 2:** Classification results of discriminant analysis of mutational profile of all 345 large B‐cell lymphoma cases based on primary organ.

Original	Predicted group (%, 95% C.I.)											
	Lymph node	NC/NPhx	Tonsil	Stomach	IC area	Other intestines	Breast	Others	Unknown	CNS	Testis	Mediastinum
Lymph node (*n* = 78)	29 (37.2, 26.5–47.9)	1	4	3	0	1	0	2	11	0	0	0
NC/NPhx (*n* = 16)	2	11 (68.8, 46.0–91.5)	1	1	0	0	0	2	9	2	0	1
Tonsil (*n* = 23)	6	1	15 (65.2, 45.8–84.7)	1	1	0	0	0	2	0	0	0
Stomach (*n* = 17)	11	0	0	11 (64.7, 42.0–87.4)	0	0	0	2	10	0	0	1
IC area (*n* = 19)	3	0	1	0	14 (73.7, 53.9–93.5)	1	0	2	8	0	0	0
Other Intestines (*n* = 12)	4	1	1	0	1	8 (66.7, 40.0–93.3)	0	0	6	1	0	0
Breast (*n* = 7)	2	0	0	0	0	0	7 (100)	0	4	3	0	0
Other sites (*n* = 30)	6	1	1	0	0	0	0	14 (46.7, 28.8–64.5)	4	0	0	1
Unknown (*n* = 94)	6	1	0	1	3	1	0	4	27 (28.7, 19.6–37.9)	1	1	0
CNS (*n* = 28)	6	0	0	0	0	0	0	4	9	18 (64.3, 46.5–82.0)	0	0
Testis (*n* = 9)	2	0	0	0	0	1	0	0	2	2	8 (88.9, 68.4–100)	0
Mediastinum (*n* = 12)	1	0	0	0	0	0	0	0	2	1	0	9 (75.0, 50.5–99.5)

Abbreviations: CNS, central nervous system; C.I., confidence interval; IC, ileocecal; NC/NPhx, nasal cavity/nasopharynx.

## 4 DISCUSSION

In this study, we analyzed the results of panel‐based NGS performed in various DLBCLs by primary organs and showed that DLBCL occurring in several specific organs had a characteristic mutational pattern. In addition to previously known information, among DLBCL of the GI tract, it was found that tumors that occurred in the IC area, stomach, and other intestines showed different characteristic mutational signatures. In our study, not only DLBCL NOS but also PCNSL, PTL, and PMLBL were included, and their mutational signatures were consistent with previously known information. PCNSL and PTL are classified together in the specific category of primary large B‐cell lymphoma of immune‐privileged sites in the WHO 5th edition^1^ and ICC.^2^ In our results, the frequency of *MYD88* ^L265P^, *PIM1*, and *CD79B* mutations and *CDKN2A*/*B* loss was significantly higher in both PCNSL and PTL than in DLBCL NOS and PMLBL, which was consistent with previous studies.^19^, ^34^, ^35^ In PMLBL, mutations in *STAT6*, *SOCS1*, *TNFAIP3*, and *B2M* and the frequency of 9p24 gain (including *CD274* and *PDCD1LG2*) were significantly higher than those in DLBCL NOS, PCNSL, and PTL, which is also consistent with the results of previous studies.^36^

Characteristic mutational patterns have been reported in DLBCL of several primary organs, which are currently classified as DLBCL NOS, and most organ‐based genetic studies have focused on the frequency of the *MYD88* ^L265P^ mutation. Some previous studies have reported high frequencies of *MYD88* ^L265P^ and *CD79B* mutations in breast DLBCL.^16^, ^17^ It is also known that the *MYD88* ^L265P^ mutation is common in extranodal DLBCL, including not only the breast but also bone, kidney/adrenal gland, testis, skin, and uterus/ovary.^11^, ^37^ However, there have been several reports that the mutation frequency of *MYD88* and *CD79B* is low in DLBCL that occurred in the GI tract, one of the extranodal sites.^18^, ^19^, ^20^ Seven cases of breast DLBCL were included in our study, and *MYD88* ^L265P^ and *CD79B* mutations were found in four and three cases, respectively, consistent with the results of previous studies. The most frequently observed mutations in breast DLBCL were *CDKN2A* and *CDKN2B* loss, which was found simultaneously in five of seven cases. *MYD88* and *CD79B* are major mutated genes in C5^9^ or MCD type^11^ DLBCL, which suggests that the majority of breast DLBCL cases belong to the C5/MCD type. In two studies on molecular classification of DLBCL, *CDKN2A*/*B* loss was suggested as a major variant gene of C2^9^ or MCD type.^11^ Considering our finding that the frequency of *CDKN2A*/*B* loss was high in PCNSL (67.9%) and PTL (55.6%), which are characterized by high frequencies of *MYD88* ^L265P^ and *CD79B* gene mutations, the authors consider that *CDKN2A*/*B* loss is likely a component of the same mutational signature that includes *MYD88* and *CD79B* mutations.

Through mutation analysis of GI tract DLBCL, we present the most novel findings in this study. The most important finding in the authors' opinion is that the stomach and IC area have a distinctly different mutational patterns from other intestinal DLBCL; therefore, they should be approached separately in each subgroup. In previous studies on GI tract DLBCL, it was concluded that the mutation frequency of *MYD88* and *CD79B* was low, without classification into specific organs.^18^, ^19^, ^20^ One study found a high frequency of *TP53* mutations in GI tract DLBCL.^20^ However, in our study, we were able to approach the mutational signature of GI DLBCL more elaborately by classifying GI tract DLBCL into the stomach, IC area, and other intestines based on a large number of samples. *MYD88* ^L265P^ and *CD79B* mutations were observed at low frequencies in all three organs in our study. *MYD88* ^L265P^ was not observed in the stomach or IC area and was observed in only 16.7% of other intestines. *CD79B* mutations were observed in 17.6%, 26.3%, and 16.7% of cases in the stomach, IC area, and other intestines, respectively. *TP53* mutation was found in 52.6% of the IC area, showing the highest frequency among all organs. The frequency of *TP53* mutations was 41.7% in other intestines and 23.5% in the stomach, which was the lowest in the GI tract. The genetic alteration most clearly showing the molecular difference between the three organs of the GI tract DLBCL was 18q21 gain. The frequency of 18q21 gain was the highest among all organs at 42.1% in the IC area, whereas it was much lower at 16.7% in the other intestines, and no 18q21 gain was observed in the stomach. In contrast, in stomach DLBCL, the mutation frequency of *PTEN* and *TNFRSF14* genes among all organs was the highest at 23.5%. *PTEN* and *TNFRSF14* mutations were not observed in the IC area. *PTEN* mutations were observed in 8.3% of other intestines, but no *TNFRSF14* mutations were observed. A previous study by our group reported that the frequency of 18q21 gain was high in intestinal DLBCL and was significantly associated with high expression of CD47.^26^ In that study, our group also used the term “intestinal type” rather than “IC area”; however, this study made it clear that the IC area DLBCL is a tumor with a different mutational signature from other intestinal DLBCL. Taken together, GI tract DLBCL had some common molecular features that differentiated it from other DLBCL NOS, but had distinct mutational signatures that differed depending on the specific locations of the stomach, IC area, and other intestines. In molecular classification studies of DLBCL, 18q21 gain has been regarded as a characteristic mutation of C5/MCD DLBCL.^9^, ^11^ In other words, it is known that 18q21 gain tends to appear together with *MYD88* and *CD79B* in immune‐privileged sites or extranodal DLBCL such as the breast. However, considering that *MYD88* ^L265P^ was not found in IC area DLBCL in our study, which had the highest frequency of 18q21 gain, it is necessary to add primary organ variables to the existing molecular subtype; thus, a more sophisticated molecular analysis of DLBCL classification is expected. Several previous studies have raised the possibility that *MYD88* mutation is a poor prognostic factor for DLBCL.^12^, ^38^ In large‐scale comprehensive molecular analysis studies, C5 and MCD were subtypes with poor prognoses.^9^, ^11^ The results of these studies are considered sufficiently meaningful, but the fact that the variable of the primary organ was not considered can be pointed out as a weakness. Additional verification is needed to determine whether the *MYD88* mutation itself is an independent prognostic factor for DLBCL under the control of primary organ variables.

In discriminant analysis to cluster and reclassify DLBCL according to the primary organ, nodal DLBCL showed the most heterogeneous mutational signature along with DLBCL of unknown origin. The special subtypes PCNSL, PTL, and PMLBL were correctly classified as the original group in most cases. However, in addition to these special subtypes, DLBCL in many specific organs was classified into the original group through discriminant analysis. Even DLBCL from other sites was reclassified as the original group in about half (46.7%), which is thought to reflect the characteristic mutational signature of extranodal DLBCL NOS. In DLBCL of all organs except lymph node and unknown organs, more than half of the cases were classified as the original group, indicating that DLBCL of each organ tends to have a unique mutational signature. However, when plotted as a scatter plot, many DLBCL NOS had centroids at similar positions. PCNSL, PTL, and PMLBL clearly demonstrated the specificity of their mutational signatures by locating the centroid distal to DLBCL NOS. Interestingly, the centroids of both organs were outside the range of the DLBCL NOS constellation. Breast DLBCL showed that the centroid was located close to PCNSL and PTL, indicating that they were relatively homogeneous C5/MCD DLBCL. IC area DLBCL had its centroid located at the most antagonistic point of immune‐privileged sites lymphoma and was outside the DLBCL NOS constellation. This suggests that IC area DLBCL is not only a homogeneous group but also a group with a unique mutational signature different from that of DLBCL NOS, like PCNSL, PTL, and PMLBL. In addition to the characteristic mutational signature of the IC area DLBCL found in this study, additional clinical, pathological, and molecular analyses are needed to investigate whether primary DLBCL of the IC area should remain a member of the DLBCL NOS or be classified as an independent subtype.

This study had several limitations. First, using panel‐based NGS, information on mutations is limited compared to those using methods such as whole genome sequencing and whole exome sequencing. In particular, the lack of information on gene translocations, such as *MYC*, *BCL2*, and *BCL6*, is a major limitation in the molecular classification of DLBCL. Second, clinical information such as prognosis was not included in the study because most of the cases were recently diagnosed and did not have a sufficient follow‐up period. Third, since the study was conducted at a single institute, the number of samples for some specific organs was insufficient, and since all cases were composed of one race, the possibility of racially specific variation could not be filtered out.

**FIGURE 4:**
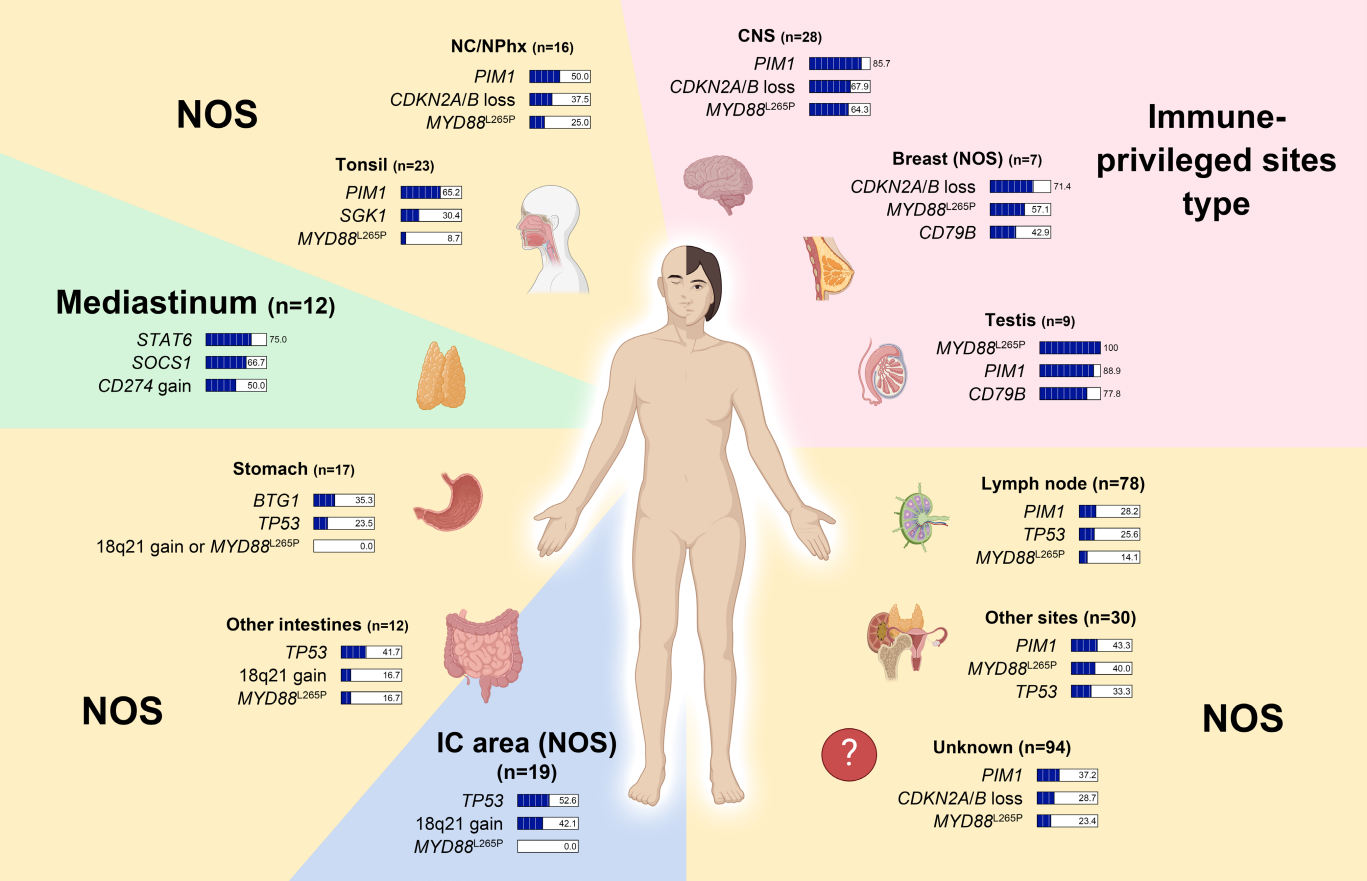
Schematic illustration showing the characteristic genetic alterations of diffuse large B‐cell lymphoma for each primary organ. Despite being diffuse large B‐cell lymphoma, not otherwise specified, tumors of the breast and ileocecal area show characteristic genetic mutation patterns (CNS, central nervous system; IC, ileocecal; NC/NPhx, nasal cavity and nasopharynx; NOS, not otherwise specified).

In conclusion, DLBCL NOS, a genetically heterogeneous entity, exhibits unique mutational signatures in some specific primary organs. The results summarizing the findings of this study are shown in Figure 4. While DLBCLs from many primary organs showed characteristic mutational profiles within the constellation of DLBCL NOS, breast DLBCL especially showed a characteristic mutational signature closer to immune‐privileged sites lymphoma than DLBCL NOS. GI tract DLBCL showed different mutational signatures in the stomach, IC area, and other intestines. Among these, IC area DLBCL showed a unique homogeneous mutational signature that was out of the constellation of DLBCL NOS. In addition to differences in mutational signatures, there is a need for further multilayered studies on the pathological and clinical differences DLBCL generated in various organs and how they can be applied to future precision medicine and targeted therapy.

## AUTHOR CONTRIBUTIONS

**Hyun Hee Koh:** Formal analysis (equal); investigation (equal); writing – original draft (lead). **Sang Eun Yoon:** Resources (equal). **Seok Jin Kim:** Resources (equal). **Won Seog Kim:** Resources (equal); supervision (equal). **Junhun Cho:** Conceptualization (lead); formal analysis (equal); investigation (equal); supervision (lead); writing – review and editing (lead).

## FUNDING INFORMATION

None.

## CONFLICT OF INTEREST STATEMENT

All authors declare no competing interests.

## Supporting information


Table S1.

Table S2.

Table S3.

## Data Availability

The datasets generated and/or analyzed during the current study are not publicly available due to the patient information security policy of our institute but are available from the corresponding author on reasonable request.
