# Supplementary material for: Differences in mutational signature of diffuse large B‐cell lymphomas according to the primary organ
Source: Cancer Med. 2023 Sep 14;12(19):19732–43. doi: 10.1002/cam4.6533 (PMC10587923; doi:10.1002/cam4.6533)
Supplement: Supplementary file 1 — Table S1. [file CAM4-12-19732-s002.docx]

**Supplementary Table 1.** The frequency of single nucleotide variants of each type of large B-cell lymphomas

|  | **All cases** | | **DLBCL NOS** | | **GCB type (NOS)** | | **non-GCB type (NOS)** | | **PCNSL** | | **PTL** | | **PMLBL** | |
| --- | --- | --- | --- | --- | --- | --- | --- | --- | --- | --- | --- | --- | --- | --- |
| 1 | *PIM1* | 39.7% | *PIM1* | 34.1% | *PIM1* | 30.9% | *PIM1* | 36.1% | *PIM1* | 85.7% | *MYD88*^L265P^ | 100.0% | *STAT6* | 75.0% |
| 2 | *CD79B* | 27.8% | *TP53* | 26.7% | *TP53* | 28.2% | *CD79B* | 29.5% | *MYD88*^L265P^ | 64.3% | *PIM1* | 88.9% | *SOCS1* | 66.7% |
| 3 | *HIST1H1E* | 25.8% | *CD79B* | 24.3% | *BTG1* | 22.7% | *TP53* | 25.7% | *CD79B* | 60.7% | *CD79B* | 77.8% | *B2M* | 58.3% |
| 4 | *TP53* | 25.2% | *BTG1* | 24.0% | *HIST1H1E* | 21.8% | *HIST1H1E* | 24.6% | *HIST1H1E* | 35.7% | *HIST1H1E* | 66.7% | *TNFAIP3* | 58.3% |
| 5 | *BTG1* | 24.9% | *HIST1H1E* | 23.6% | *EP300* | 21.8% | *ETV6* | 24.6% | *ETV6* | 35.7% | *BTG1* | 44.4% | *GNA13* | 41.7% |
| 6 | *MYD88*^L265P^ | 24.3% | *ETV6* | 20.6% | *HIST1H1C* | 20.0% | *BTG1* | 24.0% | *PRDM1* | 35.7% | *PRDM1* | 33.3% | *CIITA* | 41.7% |
| 7 | *ETV6* | 21.4% | *HIST1H1C* | 19.9% | *SGK1* | 20.0% | *MYD88*^L265P^ | 22.4% | *BTG1* | 25.0% | *TBL1XR1* | 33.3% | *PIM1* | 33.3% |
| 8 | *HIST1H1C* | 20.0% | *MYD88*^L265P^ | 19.3% | *CARD11* | 19.1% | *PRDM1* | 19.7% | *HIST1H1C* | 25.0% | *B2M* | 33.3% | *BTG1* | 33.3% |
| 9 | *PRDM1* | 19.4% | *PRDM1* | 17.9% | *SOCS1* | 18.2% | *HIST1H1C* | 19.1% | *GNA13* | 21.4% | *NOTCH2* | 33.3% | *HIST1H1D* | 33.3% |
| 10 | *B2M* | 17.4% | *CARD11* | 16.2% | *CD79B* | 16.4% | *B2M* | 16.9% | *EP300* | 17.9% | *CD58* | 22.2% | *TP53* | 33.3% |
| 11 | *CARD11* | 15.4% | *B2M* | 15.9% | *PRDM1* | 15.5% | *TBL1XR1* | 16.4% | *TBL1XR1* | 17.9% | *STAT3* | 22.2% | *HIST1H1E* | 25.0% |
| 12 | *EP300* | 14.8% | *EP300* | 15.2% | *TNFRSF14* | 15.5% | *TET2* | 15.8% | *HIST1H1D* | 17.9% | *ETV6* | 11.1% | *TBL1XR1* | 25.0% |
| 13 | *TBL1XR1* | 14.8% | *TET2* | 14.9% | *ETV6* | 14.5% | *CARD11* | 14.8% | *TP53* | 14.3% | *HIST1H1C* | 11.1% | *CD58* | 25.0% |
| 14 | *TET2* | 14.5% | *TBL1XR1* | 13.5% | *MYD88*^L265P^ | 14.5% | *TNFAIP3* | 14.8% | *CARD11* | 14.3% | *TET2* | 11.1% | *ARID1A* | 25.0% |
| 15 | *SOCS1* | 14.5% | *TNFAIP3* | 13.5% | *B2M* | 13.6% | *NOTCH1* | 14.8% | *SOCS1* | 14.3% | *BCL2* | 11.1% | *NOTCH1* | 25.0% |
| 16 | *TNFAIP3* | 14.2% | *SGK1* | 13.2% | *TET2* | 13.6% | *MEF2B* | 14.2% | *ARID1A* | 14.3% | *MEF2B* | 11.1% | *HIST1H2BC* | 25.0% |
| 17 | *NOTCH1* | 12.5% | *NOTCH1* | 12.8% | *HIST1H1D* | 13.6% | *PRKCB* | 12.6% | *MYD88*  (except L265P) | 14.3% | *BCOR* | 11.1% | *SGK1* | 25.0% |
| 18 | *SGK1* | 12.5% | *SOCS1* | 12.8% | *GNA13* | 13.6% | *EP300* | 11.5% | *B2M* | 10.7% | *CCND3* | 11.1% | *STAT3* | 16.7% |
| 19 | *CD58* | 11.6% | *MEF2B* | 11.5% | *EZH2* | 13.6% | *CD58* | 11.5% | *TET2* | 10.7% | *EZH2* | 11.1% | *ETV6* | 16.7% |
| 20 | *MEF2B* | 10.7% | *CD58* | 11.1% | *TNFAIP3* | 11.8% | *ARID1A* | 9.8% | *CIITA* | 10.7% | *TNFAIP3* | 11.1% | *HIST1H1C* | 16.7% |
| 21 | *HIST1H1D* | 10.7% | *PRKCB* | 10.5% | *FAS* | 11.8% | *BCL10* | 9.8% | *BCL2* | 10.7% | *PRKCB* | 11.1% | *TET2* | 16.7% |
| 22 | *GNA13* | 10.4% | *TNFRSF14* | 9.8% | *BCL2* | 11.8% | *NOTCH2* | 9.8% | *NOTCH1* | 7.1% | *ID3* | 11.1% | *NFKBIA* | 16.7% |
| 23 | *PRKCB* | 9.6% | *FAS* | 9.5% | *CD58* | 10.9% | *SOCS1* | 9.3% | *CD58* | 7.1% | *GNA13* | 0.0% | *PTPN1* | 16.7% |
| 24 | *ARID1A* | 9.3% | *HIST1H1D* | 9.5% | *NOTCH1* | 10.0% | *MYD88*  (except L265P) | 9.3% | *MEF2B* | 7.1% | *EP300* | 0.0% | *PRDM1* | 8.3% |
| 25 | *CIITA* | 9.3% | *MYD88*  (except L265P) | 9.1% | *MYD88*  (except L265P) | 9.1% | *SGK1* | 8.7% | *NOTCH2* | 7.1% | *HIST1H1D* | 0.0% | *NOTCH2* | 8.3% |
| 26 | *MYD88*  (except L265P) | 9.3% | *BCL10* | 8.8% | *IRF8* | 9.1% | *FAS* | 7.7% | *HIST1H2BC* | 7.1% | *TP53* | 0.0% | *CCND3* | 8.3% |
| 27 | *TNFRSF14* | 9.0% | *ARID1A* | 8.4% | *CCND3* | 9.1% | *CIITA* | 7.7% | *BCOR* | 7.1% | *CARD11* | 0.0% | *PRKCB* | 8.3% |
| 28 | *NOTCH2* | 9.0% | *GNA13* | 8.4% | *NFKBIA* | 9.1% | *HIST1H1D* | 7.1% | *CCND3* | 7.1% | *SOCS1* | 0.0% | *EP300* | 8.3% |
| 29 | *FAS* | 8.4% | *NOTCH2* | 8.4% | *CIITA* | 8.2% | *IRF8* | 7.1% | *EZH2* | 7.1% | *ARID1A* | 0.0% | *CARD11* | 8.3% |
| 30 | *BCL10* | 8.1% | *CIITA* | 8.1% | *RHOA* | 8.2% | *BCOR* | 7.1% | *BRAF* | 7.1% | *MYD88*  (except L265P) | 0.0% | *MYD88*  (except L265P) | 8.3% |
| 31 | *BCL2* | 7.8% | *BCL2* | 7.8% | *TBL1XR1* | 7.3% | *HIST1H2BC* | 7.1% | *TNFAIP3* | 3.6% | *CIITA* | 0.0% | *TNFRSF14* | 8.3% |
| 32 | *IRF8* | 7.0% | *IRF8* | 7.8% | *MEF2B* | 7.3% | *TNFRSF14* | 6.6% | *SGK1* | 3.6% | *NOTCH1* | 0.0% | *BCL10* | 8.3% |
| 33 | *HIST1H2BC* | 6.7% | *BCOR* | 6.8% | *PRKCB* | 7.3% | *GNA13* | 5.5% | *TNFRSF14* | 3.6% | *HIST1H2BC* | 0.0% | *FAS* | 8.3% |
| 34 | *BCOR* | 6.7% | *CCND3* | 6.4% | *ARID1A* | 6.4% | *BCL2* | 5.5% | *BCL10* | 3.6% | *BRAF* | 0.0% | *IRF8* | 8.3% |
| 35 | *CCND3* | 6.7% | *EZH2* | 6.1% | *BCL10* | 6.4% | *BRAF* | 5.5% | *STAT3* | 3.6% | *SGK1* | 0.0% | *MYD88*^L265P^ | 0.0% |
| 36 | *EZH2* | 6.1% | *HIST1H2BC* | 6.1% | *NOTCH2* | 6.4% | *PTEN* | 4.9% | *NFKBIA* | 3.6% | *TNFRSF14* | 0.0% | *CD79B* | 0.0% |
| 37 | *STAT3* | 5.5% | *PTEN* | 5.7% | *BCOR* | 6.4% | *CCND3* | 4.4% | *PTEN* | 3.6% | *BCL10* | 0.0% | *BCL2* | 0.0% |
| 38 | *NFKBIA* | 5.2% | *BRAF* | 5.1% | *PTEN* | 6.4% | *STAT3* | 3.8% | *STAT6* | 3.6% | *NFKBIA* | 0.0% | *MEF2B* | 0.0% |
| 39 | *PTEN* | 5.2% | *NFKBIA* | 5.1% | *STAT3* | 5.5% | *IKBKB* | 3.3% | *PRKCB* | 0.0% | *PTEN* | 0.0% | *BCOR* | 0.0% |
| 40 | *BRAF* | 4.9% | *STAT3* | 4.7% | *HIST1H2BC* | 4.5% | *NFKBIA* | 2.7% | *FAS* | 0.0% | *STAT6* | 0.0% | *EZH2* | 0.0% |
| 41 | *STAT6* | 4.9% | *RHOA* | 4.4% | *BRAF* | 4.5% | *ID3* | 2.7% | *IRF8* | 0.0% | *FAS* | 0.0% | *ID3* | 0.0% |
| 42 | *RHOA* | 3.8% | *IKBKB* | 3.7% | *IKBKB* | 4.5% | *RHOA* | 2.2% | *RHOA* | 0.0% | *IRF8* | 0.0% | *BRAF* | 0.0% |
| 43 | *IKBKB* | 3.2% | *ID3* | 2.7% | *STAT6* | 3.6% | *EZH2* | 1.6% | *IKBKB* | 0.0% | *RHOA* | 0.0% | *PTEN* | 0.0% |
| 44 | *ID3* | 2.6% | *STAT6* | 2.4% | *ID3* | 1.8% | *STAT6* | 1.6% | *ID3* | 0.0% | *IKBKB* | 0.0% | *RHOA* | 0.0% |
| 45 | *PTPN1* | 1.4% | *PTPN1* | 1.0% | *PTPN1* | 0.9% | *PTPN1* | 1.1% | *PTPN1* | 0.0% | *PTPN1* | 0.0% | *IKBKB* | 0.0% |

DLBCL, diffuse large B-cell lymphoma; NOS, not otherwise specified; GCB, germinal center B-cell; PCNSL, primary large B-cell lymphoma of the central nervous system; PTL, primary large B-cell lymphoma of the testis; PMLBL, primary mediastinal large B-cell lymphoma
