# Supplementary material for: Differences in mutational signature of diffuse large B‐cell lymphomas according to the primary organ
Source: Cancer Med. 2023 Sep 14;12(19):19732–43. doi: 10.1002/cam4.6533 (PMC10587923; doi:10.1002/cam4.6533)
Supplement: Supplementary file 2 — Table S2. [file CAM4-12-19732-s001.docx]

**Supplementary Table 2.** The frequency of copy number variations of each type of large B-cell lymphomas

|  | **All cases** | | **DLBCL NOS** | | **GCB type (NOS)** | | **non-GCB type (NOS)** | | **PCNSL** | | **PTL** | | **PMLBL** | |
| --- | --- | --- | --- | --- | --- | --- | --- | --- | --- | --- | --- | --- | --- | --- |
| 1 | *CDKN2A/B* loss | 27.8% | *CDKN2A/B* loss | 24.3% | *CDKN2A/B* loss | 21.8% | *CDKN2A/B* loss | 26.2% | *CDKN2A/B* loss | 67.9% | *CDKN2A/B* loss | 55.6% | *CD274* gain | 50.0% |
| 2 | 18q gain | 12.5% | 18q gain | 13.5% | *REL* gain | 13.6% | 18q gain | 15.8% | *PRDM1* loss | 7.1% | 18q gain | 22.2% | *PDCD1LG2* gain | 41.7% |
| 3 | *REL* gain | 5.5% | *REL* gain | 6.4% | 18q gain | 10.0% | *CD274* gain | 3.8% | 18q gain | 3.6% | *PRDM1* loss | 0.0% | *CD58* loss | 8.3% |
| 4 | *CD274* gain | 4.1% | *CD274* gain | 2.4% | *CD58* loss | 2.7% | *PDCD1LG2* gain | 2.7% | *CD274* gain | 3.6% | *CD274* gain | 0.0% | *CDKN2A/B* loss | 0.0% |
| 5 | *PDCD1LG2* gain | 3.2% | *B2M* loss | 1.7% | *B2M* loss | 1.8% | *REL* gain | 2.2% | *PDCD1LG2* gain | 3.6% | *PDCD1LG2* gain | 0.0% | 18q gain | 0.0% |
| 6 | *B2M* loss | 1.7% | *PDCD1LG2* gain | 1.7% | *TNFRSF14* loss | 1.8% | *B2M* loss | 1.6% | *B2M* loss | 3.6% | *B2M* loss | 0.0% | *PRDM1* loss | 0.0% |
| 7 | *CD58* loss | 1.4% | *CD58* loss | 1.4% | *PTEN* loss | 0.9% | *PRDM1* loss | 1.6% | *REL* gain | 0.0% | *REL* gain | 0.0% | *B2M* loss | 0.0% |
| 8 | *PRDM1* loss | 1.4% | *PRDM1* loss | 1.0% | *CD274* gain | 0.0% | *CD58* loss | 0.5% | *CD58* loss | 0.0% | *CD58* loss | 0.0% | *REL* gain | 0.0% |
| 9 | *TNFRSF14* loss | 0.9% | *TNFRSF14* loss | 1.0% | *PDCD1LG2* gain | 0.0% | *TNFRSF14* loss | 0.5% | *TNFRSF14* loss | 0.0% | *TNFRSF14* loss | 0.0% | *TNFRSF14* loss | 0.0% |
| 10 | *PTEN* loss | 0.6% | *PTEN* loss | 0.7% | *PRDM1* loss | 0.0% | *PTEN* loss | 0.5% | *PTEN* loss | 0.0% | *PTEN* loss | 0.0% | *PTEN* loss | 0.0% |
| 11 | *RB1* loss | 0.3% | *RB1* loss | 0.3% | *RB1* loss | 0.0% | *RB1* loss | 0.5% | *RB1* loss | 0.0% | *RB1* loss | 0.0% | *RB1* loss | 0.0% |

DLBCL, diffuse large B-cell lymphoma; NOS, not otherwise specified; GCB, germinal center B-cell; PCNSL, primary large B-cell lymphoma of the central nervous system; PTL, primary large B-cell lymphoma of the testis; PMLBL, primary mediastinal large B-cell lymphoma
