# Supplementary material for: Differences in mutational signature of diffuse large B‐cell lymphomas according to the primary organ
Source: Cancer Med. 2023 Sep 14;12(19):19732–43. doi: 10.1002/cam4.6533 (PMC10587923; doi:10.1002/cam4.6533)
Supplement: Supplementary file 3 — Table S3. [file CAM4-12-19732-s003.docx]

**Supplementary Table 3.** The frequency of genetic alterations of diffuse large B-cell lymphoma, not otherwise specified cases according to primary organ

| **Gene** | **LN** | **NC/NPhx** | **Tonsil** | **Stomach** | **IC area** | **Other  intestines** | **Breast** | **Others** | **Unknown** |
| --- | --- | --- | --- | --- | --- | --- | --- | --- | --- |
| *ARID1A* | 9.0% | 6.2% | 4.3% | 0.0% | 10.5% | 0.0% | 28.6% | 3.3% | 11.7% |
| *B2M* | 17.9% | 6.2% | 13.0% | 11.8% | 26.3% | 33.3% | 42.9% | 16.7% | 10.6% |
| *BCL10* | 11.5% | 6.2% | 8.7% | 17.6% | 0.0% | 0.0% | 0.0% | 6.7% | 9.6% |
| *BCL2* | 6.4% | 0.0% | 4.3% | 0.0% | 10.5% | 0.0% | 0.0% | 10.0% | 12.8% |
| *BCOR* | 9.0% | 0.0% | 0.0% | 17.6% | 0.0% | 0.0% | 14.3% | 10.0% | 6.4% |
| *BRAF* | 2.6% | 0.0% | 0.0% | 5.9% | 10.5% | 16.7% | 14.3% | 3.3% | 6.4% |
| *BTG1* | 28.2% | 31.2% | 30.4% | 35.3% | 5.3% | 0.0% | 0.0% | 23.3% | 24.5% |
| *CARD11* | 12.8% | 6.2% | 26.1% | 29.4% | 15.8% | 25.0% | 28.6% | 13.3% | 14.9% |
| *CCND3* | 10.3% | 6.2% | 0.0% | 5.9% | 0.0% | 0.0% | 14.3% | 6.7% | 6.4% |
| *CD58* | 14.1% | 6.2% | 4.3% | 0.0% | 5.3% | 8.3% | 0.0% | 16.7% | 13.8% |
| *CD79B* | 23.1% | 18.8% | 21.7% | 17.6% | 26.3% | 16.7% | 42.9% | 30.0% | 25.5% |
| *CIITA* | 10.3% | 6.2% | 4.3% | 11.8% | 5.3% | 0.0% | 42.9% | 3.3% | 7.4% |
| *EP300* | 7.7% | 18.8% | 0.0% | 17.6% | 21.1% | 25.0% | 28.6% | 13.3% | 21.3% |
| *ETV6* | 20.5% | 31.2% | 21.7% | 11.8% | 5.3% | 0.0% | 28.6% | 16.7% | 26.6% |
| *EZH2* | 5.1% | 0.0% | 0.0% | 0.0% | 5.3% | 8.3% | 0.0% | 6.7% | 10.6% |
| *FAS* | 11.5% | 0.0% | 8.7% | 23.5% | 10.5% | 0.0% | 0.0% | 13.3% | 7.4% |
| *GNA13* | 9.0% | 0.0% | 17.4% | 5.9% | 0.0% | 8.3% | 14.3% | 10.0% | 8.5% |
| *HIST1H1C* | 15.4% | 12.5% | 17.4% | 23.5% | 21.1% | 8.3% | 14.3% | 23.3% | 25.5% |
| *HIST1H1D* | 11.5% | 6.2% | 13.0% | 17.6% | 0.0% | 0.0% | 0.0% | 10.0% | 9.6% |
| *HIST1H1E* | 24.4% | 25.0% | 21.7% | 23.5% | 21.1% | 16.7% | 14.3% | 26.7% | 24.5% |
| *HIST1H2BC* | 3.8% | 25.0% | 0.0% | 5.9% | 5.3% | 0.0% | 0.0% | 6.7% | 7.4% |
| *ID3* | 3.8% | 0.0% | 0.0% | 5.9% | 0.0% | 0.0% | 0.0% | 3.3% | 3.2% |
| *IKBKB* | 3.8% | 6.2% | 0.0% | 0.0% | 10.5% | 0.0% | 0.0% | 3.3% | 4.3% |
| *IRF8* | 7.7% | 6.2% | 0.0% | 5.9% | 15.8% | 0.0% | 0.0% | 13.3% | 8.5% |
| *MEF2B* | 11.5% | 12.5% | 13.0% | 0.0% | 15.8% | 8.3% | 14.3% | 16.7% | 10.6% |
| *MYD88*^L265P^ | 14.1% | 25.0% | 8.7% | 0.0% | 0.0% | 16.7% | 57.1% | 40.0% | 23.4% |
| *MYD88*  (except L265P) | 6.4% | 25.0% | 13.0% | 5.9% | 0.0% | 16.7% | 0.0% | 6.7% | 10.6% |
| *NFKBIA* | 2.6% | 0.0% | 13.0% | 0.0% | 5.3% | 16.7% | 0.0% | 10.0% | 4.3% |
| *NOTCH1* | 14.1% | 18.8% | 8.7% | 5.9% | 15.8% | 8.3% | 14.3% | 10.0% | 13.8% |
| *NOTCH2* | 14.1% | 12.5% | 4.3% | 5.9% | 0.0% | 16.7% | 0.0% | 10.0% | 5.3% |
| *PIM1* | 28.2% | 50.0% | 65.2% | 11.8% | 10.5% | 8.3% | 42.9% | 43.3% | 37.2% |
| *PRDM1* | 16.7% | 25.0% | 8.7% | 17.6% | 10.5% | 8.3% | 14.3% | 26.7% | 20.2% |
| *PRKCB* | 9.0% | 12.5% | 8.7% | 23.5% | 10.5% | 0.0% | 14.3% | 10.0% | 10.6% |
| *PTEN* | 7.7% | 0.0% | 0.0% | 23.5% | 0.0% | 8.3% | 0.0% | 3.3% | 5.3% |
| *PTPN1* | 2.6% | 0.0% | 0.0% | 0.0% | 0.0% | 0.0% | 0.0% | 0.0% | 1.1% |
| *RHOA* | 2.6% | 6.2% | 0.0% | 5.9% | 5.3% | 8.3% | 0.0% | 10.0% | 4.3% |
| *SGK1* | 14.1% | 18.8% | 30.4% | 23.5% | 10.5% | 16.7% | 14.3% | 6.7% | 7.4% |
| *SOCS1* | 16.7% | 12.5% | 30.4% | 5.9% | 0.0% | 8.3% | 14.3% | 6.7% | 11.7% |
| *STAT3* | 2.6% | 6.2% | 8.7% | 5.9% | 5.3% | 16.7% | 0.0% | 3.3% | 4.3% |
| *STAT6* | 3.8% | 0.0% | 0.0% | 0.0% | 0.0% | 0.0% | 14.3% | 0.0% | 3.2% |
| *TBL1XR1* | 7.7% | 25.0% | 13.0% | 11.8% | 0.0% | 0.0% | 28.6% | 16.7% | 19.1% |
| *TET2* | 12.8% | 6.2% | 8.7% | 17.6% | 10.5% | 16.7% | 14.3% | 13.3% | 20.2% |
| *TNFAIP3* | 17.9% | 6.2% | 17.4% | 17.6% | 10.5% | 8.3% | 14.3% | 16.7% | 9.6% |
| *TNFRSF14* | 14.1% | 0.0% | 4.3% | 23.5% | 0.0% | 0.0% | 0.0% | 16.7% | 8.5% |
| *TP53* | 25.6% | 18.8% | 13.0% | 23.5% | 52.6% | 41.7% | 0.0% | 33.3% | 25.5% |
| *B2M* loss | 1.3% | 0.0% | 0.0% | 0.0% | 0.0% | 0.0% | 0.0% | 6.7% | 2.1% |
| *BCL2* gain | 6.4% | 0.0% | 4.3% | 0.0% | 15.8% | 8.3% | 0.0% | 3.3% | 5.3% |
| *CD274* gain | 1.3% | 6.2% | 0.0% | 0.0% | 0.0% | 0.0% | 0.0% | 0.0% | 5.3% |
| *CD58* loss | 1.3% | 0.0% | 0.0% | 5.9% | 0.0% | 0.0% | 0.0% | 3.3% | 1.1% |
| *CDKN2A* loss | 15.4% | 25.0% | 13.0% | 17.6% | 10.5% | 8.3% | 71.4% | 23.3% | 25.5% |
| *CDKN2B* loss | 16.7% | 37.5% | 13.0% | 11.8% | 5.3% | 8.3% | 71.4% | 16.7% | 22.3% |
| *MALT1* gain | 7.7% | 6.2% | 8.7% | 0.0% | 36.8% | 8.3% | 0.0% | 6.7% | 6.4% |
| *PDCD1LG2* gain | 2.6% | 0.0% | 0.0% | 0.0% | 0.0% | 0.0% | 0.0% | 0.0% | 3.2% |
| *PRDM1* loss | 3.8% | 0.0% | 0.0% | 0.0% | 0.0% | 0.0% | 0.0% | 0.0% | 0.0% |
| *PTEN* loss | 1.3% | 0.0% | 4.3% | 0.0% | 0.0% | 0.0% | 0.0% | 0.0% | 0.0% |
| *RB1* loss | 0.0% | 0.0% | 0.0% | 0.0% | 0.0% | 0.0% | 0.0% | 0.0% | 1.1% |
| *REL* gain | 7.7% | 6.2% | 13.0% | 0.0% | 15.8% | 0.0% | 0.0% | 6.7% | 4.3% |
| *SMAD2* gain | 12.8% | 12.5% | 13.0% | 0.0% | 26.3% | 8.3% | 14.3% | 16.7% | 6.4% |
| *SMAD4* gain | 9.0% | 0.0% | 4.3% | 0.0% | 26.3% | 8.3% | 14.3% | 10.0% | 4.3% |
| *TNFRSF11A* gain | 6.4% | 0.0% | 4.3% | 0.0% | 26.3% | 0.0% | 14.3% | 3.3% | 4.3% |
| *TNFRSF14* loss | 2.6% | 0.0% | 0.0% | 0.0% | 0.0% | 0.0% | 0.0% | 0.0% | 1.1% |

LN, lymph node; NC/NPhx, nasal cavity/nasopharynx; IC, ileocecal
